# NHERF1 together with PARP1 and BRCA1 expression as a new potential biomarker to stratify breast cancer patients

**DOI:** 10.18632/oncotarget.19444

**Published:** 2017-07-22

**Authors:** Anita Mangia, Emanuela Scarpi, Giulia Partipilo, Laura Schirosi, Giuseppina Opinto, Francesco Giotta, Giovanni Simone

**Affiliations:** ^1^ Functional Biomorphology Laboratory, IRCCS-Istituto Tumori “Giovanni Paolo II”, Bari 70124, Italy; ^2^ Unit of Biostatistics and Clinical Trials, (IRST)-IRCCS-Istituto Scientifico Romagnolo per lo Studio e la Cura dei Tumori, Meldola (FC) 47014, Italy; ^3^ Medical Oncology Unit, IRCCS-Istituto Tumori “Giovanni Paolo II”, Bari 70124, Italy; ^4^ Pathology Department, IRCCS-Istituto Tumori “Giovanni Paolo II”, Bari 70124, Italy

**Keywords:** Na^+^/H^+^ exchanger regulatory factor 1 (NHERF1), PARP1, BRCA1, breast cancer, immunohistochemistry

## Abstract

It has been recognized that Na^+^/H^+^ Exchanger Regulatory Factor 1 (NHERF1) in breast cancer (BC) acts as a tumor suppressor or as an oncogenic protein, depending on its subcellular localization. This study aims to correlate NHERF1 expression to BRCA1 and PARP1 proteins, to investigate their relationship, and their biological and clinical significance. Using immunohistochemistry on tissue microarrays, we evaluated subcellular NHERF1, BRCA1 and PARP1 expression in 308 BCs including a subgroup (n=80) of triple negative BCs (TNBCs). Herein, we show that nuclear NHERF1 (nNHERF1) expression was significantly associated with nuclear BRCA1 (nBRCA1) expression (*p*=0.0008), and an association was also found between nuclear PARP1 (nPARP1) and nBRCA1 (*p*<0.0001). Cytoplasmic NHERF1 (cNHERF1) was correlated to nPARP1 (*p*<0.0001). Survival analyses showed that the patients with positive nPARP1 and nNHERF1 tended toward a shorter 5-year overall survival (OS) (*p*=0.057). In TNBCs, the association between nBRCA1 and nPARP1 was maintained (*p*<0.0001), and an association between nNHERF1 and nPARP1 was observed (*p*=0.010). Univariate analysis revealed that TNBCs with positive cNHERF1 and nPARP1 had a shorter 5-year OS (*p*=0.048).

Our data suggest that NHERF1 could be a new potential biomarker in combination with PARP1 and BRCA1 expression to stratify BC patients. In particular, in TNBCs, cNHERF1 associated with nPARP1 expression identified a patient subgroup with a shorter survival, for whom it may be useful to develop novel therapeutic strategies.

## INTRODUCTION

Breast cancer (BC) remains the most common malignancy in women in many countries [1] and is the leading cause of cancer death, despite great advances in early diagnosis and treatments. Numerous factors, both genetic and non-genetic, have been well documented as regards the aetiology of breast cancer, but it is not possible to identify specific risk factors [2]. The different clinical outcome and response to therapy of BC patients depend on the stage of disease, the biomolecular complexity and pathological features of this tumor. Understanding the expression of the molecules involved in cell signaling, control of cell growth, DNA repair and death, could improve knowledge of the pathways that contribute to the cancerogenesis, tumor differentiation and progression of breast cancer.

Na^+^/H^+^ Exchanger Regulatory Factor1/ezrin-radixin-moesin (ERM) binding phosphoprotein of 50 KDa (NHERF1/EBP50), which is a scaffold multifunctional protein, binds ERM proteins through its C-terminal ezrin-binding domain, a large variety of other proteins via its two tandem PDZ (postsynaptic density 95/disc-large/zona) domains, and many cancer-related proteins [3-6].

The NHERF1 protein is physiologically expressed at the apical membrane of polarized epithelial cells and it is a major component of signalling complexes [7, 8]. Increased NHERF1 expression has been reported in a variety of human malignant tissues [3, 9-14]. The most extensive analyses of the role of NHERF1 in cancer development have been performed for BC [7, 15-17]. In tumors, the expression and different subcellular distribution of NHERF1 (from the apical membrane to the cytoplasm or nucleus) is compatible with its dual role [18, 19]. NHERF1 may behave either as a tumor suppressor [12, 20-22] when it is localized at the plasma membrane, or as an oncogenic protein [3, 10, 17, 23] when it is shifted to the cytoplasm or nucleus. Also in breast tumors, a different biological significance of the subcellular localization of this protein has been observed. In particular, cytoplasmic overexpression of NHERF1 is related to features of aggressive behaviour, such as negative hormonal status, high proliferative activity, epidermal growth factor receptor 2 (HER2) positive tumors and poor outcome [23]. Nuclear NHERF1 expression was associated with small tumor size and positive hormonal status. Furthermore, the loss of NHERF1 nuclear expression is associated with unfavourable prognosis [23, 24]. Moreover, we observed that the progressive cytoplasmic NHERF1 overexpression and the decrease of membranous NHERF1 expression were related to BC development and progression, suggesting an important role for this protein during the carcinogenesis [19]. It is known that carcinogenesis and tumor growth are influenced by various factors that cause DNA damage [25]. Molecular genetic studies have shown the involvement of several genes in DNA damage response (DDR) [25]. Different DNA repair pathways are specific for each different set of DNA lesions. DDR is accomplished through the combination of an intricate pool of proteins and when one of the mechanisms is inefficient, some others prosper, spinning the DNA repair towards another pathway. Even if the alternate mechanism is damaged, genetic instability occurs and leads to cell death [26]. DNA repair pathways play key roles in maintaining genomic stability and influence carcinogenesis and tumor biology. The knowledge of the damaged pathway can help not only to understand the interaction between the various DNA repair systems but also to find potential candidate targets for selective therapy.

It is well known that DNA repair deficiencies are risk factors for a variety of malignancies [27], including BC. A number of reports in the literature have demonstrated that dysfunction of the tumor suppressor genes, either BRCA1 or BRCA2, is synthetically lethal, with inhibition of the DNA repair enzyme Poly[ADP-Ribose] Polymerase 1 (PARP1) [28].

PARP1, the most abundant member of the PARP superfamily, is a key DNA repair factor involved in base excision repair occurring in response to DNA damage. It is a highly conserved cell signalling protein that exclusively catalyses poly ADP-ribosylation of DNA-binding proteins, such as BRCA1, thereby modulating their activity. Although overexpression of PARP1 is found in different primary human tumors [29-34], the biological and clinical significance of the protein in breast cancer has yet to be fully elucidated. The tumor suppressor BRCA1 is among the central components of the surveillance system and it recruits various DNA repair proteins to the sites of damage. It ensures high-fidelity double-strand break DNA repair, maintaining genomic stability by homologous recombination [35]. Functional-loss mutations in the BRCA1 gene lead to genome instability and predispose to familial BC. Epigenetic silencing, which disrupts BRCA1 transcriptional activity, can also be decisive for tumor formation in sporadic BCs [36]. Recently, we demonstrated that nuclear PARP1 expression was significantly associated with BRCA1 expression [37, 38], underlining the fact that the two DNA repair pathways can be contextually up-regulated.

In this study, we evaluated NHERF1, BRCA1 and PARP1 protein expression by means of immuno-histochemistry in a retrospective series of invasive BC including a subgroup of triple negative breast cancers (TNBCs). The purpose was to correlate, for the first time, the different subcellular distribution of NHERF1 to BRCA1 and PARP1 expression. Moreover, we also assessed the expression pattern of the proteins in relation to BC clinicopathological characteristics and patient outcome, in order to investigate their biological and clinical significance.

## RESULTS

The clinicopathological characteristics of the 308 BC patients included in this study are summarized in Supplementary Table 1. A total of 48.7% of the patients were older than 51 years. The majority of the patients had invasive ductal carcinoma (IDC) (87%), a moderate histological grade (G2) (47.4%), and were equal or smaller than 2 cm (53.8%) with no axillary lymph node involvement (57.2%). The majority had estrogen receptor (ER) positive tumors (64.2%), progesterone receptor (PgR) positive tumors (52.1%), high proliferative activity (Ki67 index) (52.0%) and HER2/neu negative tumors (81.7 %).

### Expression of NHERF1, BRCA1, PARP1 in invasive BC

**Figure 1:**
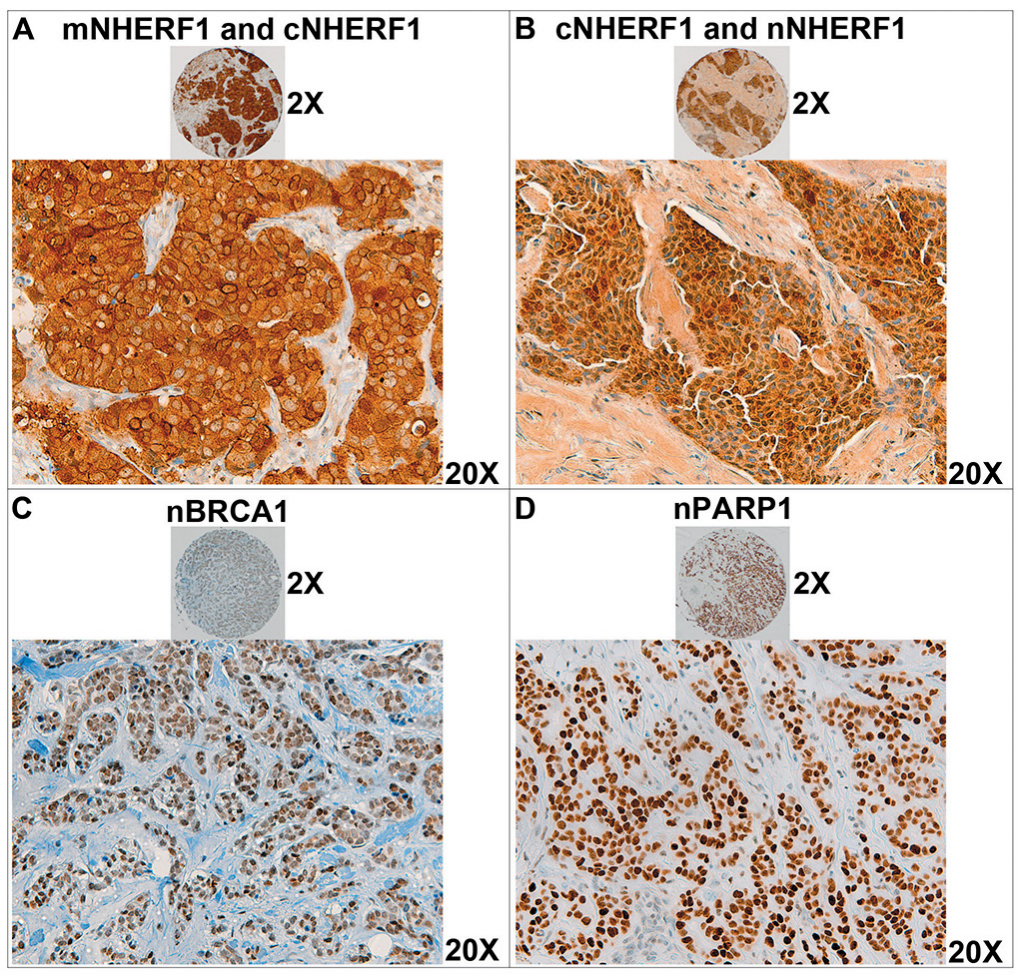
Immunoreactivity of NHERF1, BRCA1 and PARP1 proteins on breast cancer tissue microarrays (TMA) Representative images of immunohistochemical staining of TMA tumor cores for NHERF1, BRCA1 and PARP1 proteins. **(A)** Invasive breast tumor cells with high membranous NHERF1 and cytoplasmic NHERF1 expression. **(B)** A tumor sample with nuclear NHERF1 and cytoplasm NHERF1 expression. **(C)** A breast tumor TMA core showing higher nuclear BRCA1 expression. **(D)** A breast tumor sample with positive nuclear PARP1 expression. Panoramic views of the tumor cores at original magnification 2X (upper) and detail views at original magnification 20X (down).

The negative and positive expression of the proteins according to the cut-off, are described in the Material and Methods section. NHERF1 expression was detected in the apical membrane, cytoplasm, and nucleus of 281/308 (91.2%) tumor cases. Membranous NHERF1 (mNHERF1) was positive in 24.2% (68/281) of cases, cytoplasmic NHERF1 (cNHERF1) was positive in 56.2% (158/281) of samples, and nuclear NHERF1 (nNHERF1) was positive in 16.4% (46/281) of cases. Nuclear BRCA1 (nBRCA1) expression was assessed in 256/308 (83.1%) of tumors and it was positive in 131/256 (51.2%) of cases. Nuclear PARP1 (nPARP1) expression was assessed in 263/308 (85.4%) of tumor samples. nPARP1 expression was positive in 76/263 (28.9%) of tumor cases. Figure 1(A-D) shows some examples of mNHERF1, cNHERF1, nNHERF1, nBRCA1 and nPARP1 immunohistochemical staining patterns in tissue microarray (TMA) tumor cores.

### Association between protein expressions

We first analysed the association between the tumor biomarkers. Considering the dichotomized variables, nNHERF1 expression was significantly associated with nBRCA1 expression (*p*=0.0008) and there was a significant association between nPARP1 and nBRCA1 expression (*p*<0.0001) (Table 1). The statistical analyses, using continuous variables (% of expression of the proteins), showed that nNHERF1 expression was directly correlated to nBRCA1 expression (r_s_=0.21; *p*=0.001) and a direct correlation was also found between cNHERF1 and nPARP1 expression (r_s_=0.30; *p*<0.0001). Moreover, nPARP1 was significantly associated with nBRCA1 expression (r_s_=0.41; *p*<0.0001).

**Table 1:** Association between protein expressions

	*mNHERF1*			*cNHERF1*			*nNHERF1*			*nBRCA1*		
	Negative	Positive	*p-value*	Negative	Positive	*p-value*	Negative	Positive	*p-value*	Negative	Positive	*p-value*
	n (%)	n (%)		n (%)	n (%)		n (%)	n (%)		n (%)	n (%)	
cNHERF1												
Negative	95 (44.6)	28 (41.2)	0.621									
Positive	118 (55.4)	40 (58.8)										
nNHERF1												
Negative	176 (82.6)	59 (86.8)	0.423	100 (81.3)	135 (85.4)	0.353						
Positive	37 (17.4)	9 (13.2)		23 (18.7)	23 (14.6)							
nBRCA1												
Negative	93 (48.9)	30 (48.4)	0.939	55 (50.5)	68 (47.5)	0.648	112 (53.6)	11 (25.6)				
Positive	97 (51.1)	32 (51.6)		54 (49.5)	75 (52.5)		97 (46.4)	32 (74.4)	0.0008			
nPARP1												
Negative	136 (70.1)	47 (74.6)	0.494	84 (76.4)	99 (67.4)	0.115	154 (72.0)	29 (67.4)	0.551	101 (85.6)	72 (56.7)	<0.0001
Positive	58 (29.9)	16 (25.4)		26 (23.6)	48 (32.6)		60 (28.0)	14 (32.6)		17 (14.4)	55 (43.3)	

*p-value* by Chi-square or Fisher Test. Bold values indicate significance.

mNHERF1 membranous NHERF1, cNHERF1 cytoplasmic NHERF1, nNHERF1 nuclear NHERF1, nBRCA1 nuclear BRCA1, nPARP1 nuclear PARP1

### Association between protein expression and clinicopathological characteristics

Supplementary Table 2 shows the association among mNHERF1, cNHERF1, nNHERF1, nBRCA1 and nPARP1 protein expression and the clinicopathological characteristics. Positive mNHERF1 expression was associated with ER-positive (*p*=0.0001) and PgR-positive (*p*=0.0040) status. An inverse association was observed between mNHERF1 and Ki67 index (*p*=0.035). Moreover, positive mNHERF1 expression was weakly associated with negative HER2/neu status (*p*=0.0500). Statistical analyses showed that positive cNHERF1 expression was associated with patient age (*p*=0.0009), negative lymph node status (*p*=0.0470) and high histological grade (*p*=0.0460). Analysis of the clinicopathological significance of nBRCA1 and nPARP1 expression revealed that nBRCA1 expression was significantly associated with HER2/neu status (*p*=0.035) and that positive nPARP1 expression was associated with IDC (*p*=0.006).

### Expression of the proteins and patient outcome

**Figure 2:**
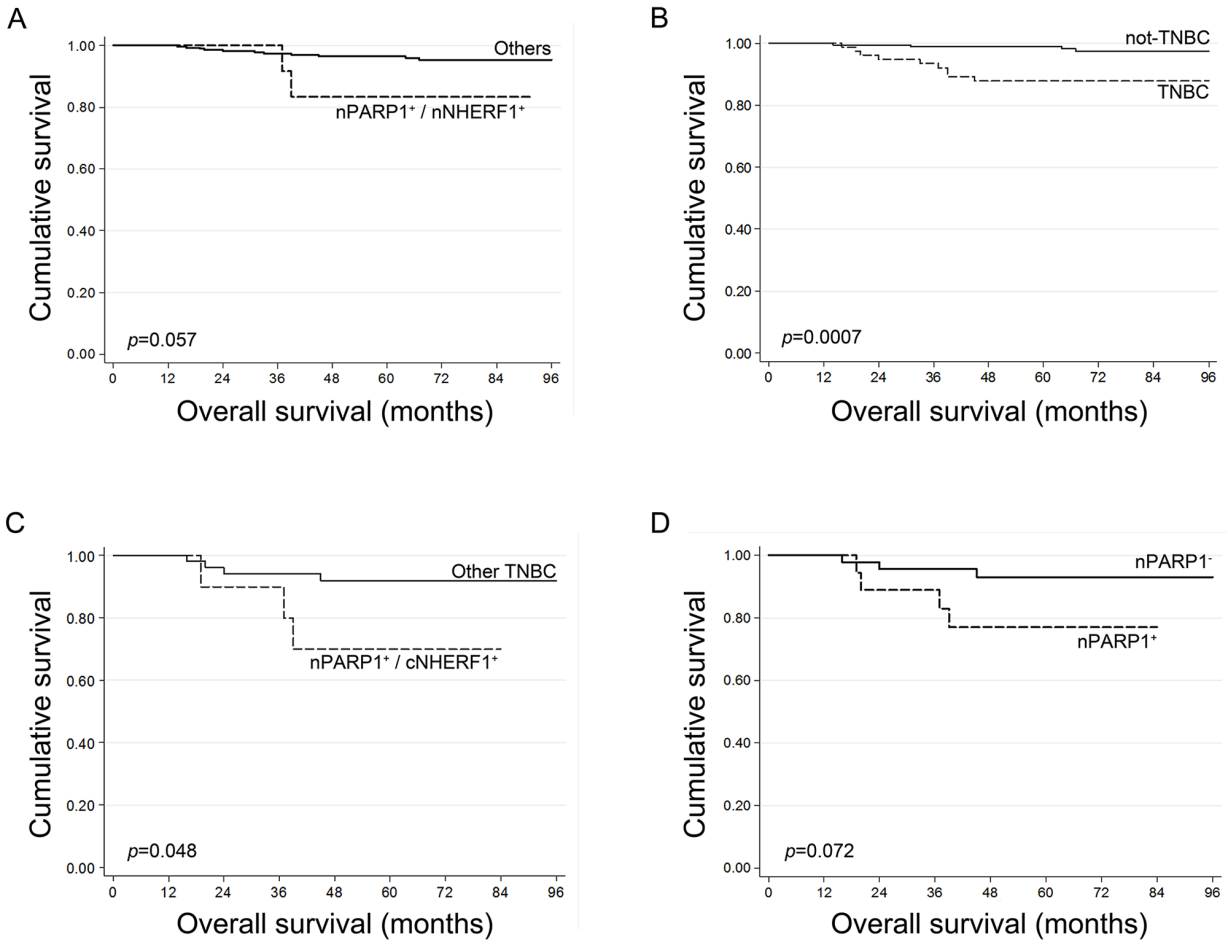
Kaplan-Meier survival curves **(A)** Overall survival according to nPARP1 and nNHERF1 expression in the 308 patients. **(B)** Overall survival of TNBC patients respect to not-TNBC patients. **(C)** Overall survival according to nPARP1 and cNHERF1 expression in TNBC patients. **(D)** Overall survival according to nPARP1 expression in TNBC patients.

Univariate and multivariate survival analyses were carried out including all the clinicopathological characteristics and the expression of mNHERF1, cNHERF1, nNHERF1, nPARP1 and nBRCA1 proteins, as dichotomized variables, and were correlated to disease-free survival (DFS) and overall-survival (OS). No significant differences were observed in the DFS and in the OS analyses between patients with high and low mNHERF1, high and low cNHERF1, high and low nNHERF1, high and low nBRCA1 and with high and low nPARP1 expression (Table 2). Regarding the analysed proteins, we found only that the subgroup of patients with positive expression of nPARP1 and nNHERF1 had a trend toward a shorter 5-year OS, 83% vs 97% for the other patients (*p*=0.057) (Figure 2A). Moreover, univariate analysis of clinicopathological characteristics in the entire cohort revealed that high histological grade (*p*=0.014), positive lymph nodes (*p*=0.007), large tumor size (*p*=0.028), and positive Ki67 index (*p*=0.004) were significantly associated with worse DFS. In the univariate analysis for OS, high histological grade (*p*=0.012), ER-negative expression (*p*=0.008), PgR-negative expression (*p*=0.022) and positive Ki67 status (*p*=0.024) were significantly associated with worse OS (Table 2). When using continuous data, univariate Cox regression analysis showed that tumor size (*p*=0.011) and Ki67 index (*p*=0.028) were significantly associated with poor DFS, while ER (*p*=0.016), PgR (*p*=0.045) and Ki67 index (*p*=0.012) were associated with OS (Table 3).

**Table 2:** Univariate analysis with respect to DFS and OS in 308 patients with invasive breast cancer

		DFS				OS			
	No. of pts	No. of events	5-year DFS (95% CI)^1^	HR (95% CI)^2^	*p-value*^2^	No. of events	5-year OS (95% CI)^1^	HR (95% CI)^2^	*p-value*^2^
Overall	308	55	87 (83-91)	-	-	13	96 (93-98)	-	-
mNHERF1									
Negative (=0)	213	43	85 (80-90)	1.00		11	95 (91-98)	1.00	
Positive (>0)	68	10	91 (84-99)	0.81 (0.40-1.62)	0.552	2	98 (95-100)	0.54 (0.12-2.45)	0.428
cNHERF1									
Negative (<60)	123	29	87 (80-93)	1.00		6	96 (92-99)	1.00	
Positive (≥60)	158	24	87 (81-93)	0.76 (0.44-1.33)	0.340	7	95 (92-99)	1.04 (0.35-3.11)	0.941
nNHERF1									
Negative (=0)	235	46	86 (81-91)	1.00		9	96 (94-99)	1.00	
Positive (>0)	46	7	90 (81-99)	0.68 (0.30-1.51)	0.345	4	93 (84-100)	2.11 (0.65-6.86)	0.214
nBRCA1									
Negative (<3)	125	20	89 (83-95)	1.00		3	98 (96-100)	1.00	
Positive (≥3)	131	24	90 (84-95)	1.28 (0.70-2.35)	0.416	7	95 (90-99)	2.27 (0.59-8.80)	0.234
nPARP1									
Negative (0≤QS≤9)	187	35	86 (81-92)	1.00		7	97 (94-100)	1.00	
Positive (10≤QS≤18)	76	12	91 (84-98)	1.03 (0.53-2.00)	0.925	4	93 (87-100)	1.58 (0.46-5.41)	0.466
Histological grade									
G1+G2	166	20	93 (89-98)	1.00		2	99 (86-100)	1.00	
G3	138	34	80 (73-87)	2.00 (1.15-3.49)	0.014	11	92 (87-97)	6.83 (1.51-30.81)	0.012
Lymph node status									
Negative	174	21	91 (86-96)	1.00		7	96 (93-99)	1.00	
Positive	130	31	84 (77-91)	2.15 (1.24-3.75)	0.007	6	95 (91-99)	1.20 (0.40-3.59)	0.737
Tumor size (cm)									
≤2 cm	156	18	95 (91-99)	1.00		5	97 (94-100)	1.00	
>2 cm	134	31	80 (73-88)	1.92 (1.07-3.43)	0.028	6	96 (92-99)	1.36 (0.41-4.45)	0.612
ER									
Negative (≤10%)	110	23	82 (74-89)	1.00		10	90 (84-96)	1.00	
Positive (>10%)	197	31	91 (86-95)	0.91 (0.53-1.57)	0.738	3	99 (98-100)	0.17 (0.05-0.64)	0.008
PgR									
Negative (≤10%)	147	27	84 (78-91)	1.00		11	92 (88-97)	1.00	
Positive (>10%)	160	27	90 (85-95)	1.02 (0.60-1.75)	0.934	2	99 (98-100)	0.17 (0.04-0.78)	0.022
Ki67 index									
Negative (≤20%)	147	13	93 (89-98)	1.00		1	99 (98-100)	1.00	
Positive (>20%)	159	41	82 (76-88)	2.49 (1.33-4.66)	0.004	12	93 (89-97)	10.40 (1.35-80.01)	0.024
HER2/neu									
Negative (0,1+)	241	43	88 (83-92)	1.00		12	95 (92-98)	1.00	
Positive (3+)	54	7	86 (75-97)	0.73 (0.33-1.63)	0.445	1	98 (94-100)	0.42 (0.05-3.24)	0.406

^1^Kaplan-Meier methods, ^l^ logrank test, ^2^Cox regression model. Bold values indicate significance.

mNHERF1 membranous NHERF1, cNHERF1 cytoplasmic NHERF1, nNHERF1 nuclear NHERF1, nBRCA1 nuclear BRCA1, nPARP1 nuclear PARP1, ER estrogen receptor, PgR progesterone receptor, DFS disease free survival, OS overall survival, HR hazard ratio, CI confidence interval

**Table 3:** Univariate analysis with respect to DFS and OS in 308 patients with breast cancer

	DFS		OS	
	HR (95% CI)^1^	p^1^	HR (95% CI)^1^	p^1^
mNHERF1	0.99 (0.97-1.01)	0.316	1.00 (0.97-1.03)	0.785
cNHERF1	0.99 (0.99-1.01)	0.341	0.99 (0.97-1.01)	0.275
nNHERF1	0.98 (0.95-1.02)	0.274	0.97 (0.89-1.06)	0.533
nBRCA1	0.9 (0.97-1.01)	0.501	0.92 (0.82-1.03)	0.157
nPARP1	1.00 (0.99-1.01)	0.607	0.99 (0.97-1.02)	0.689
Tumor size (cm)	1.26 (1.05-1.51)	0.011	1.36 (0.97-1.92)	0.073
ER	1.00 (0.99-1.01)	0.613	0.97 (0.95-0.99)	0.016
PgR	1.00 (0.99-1.01)	0.530	0.96 (0.93-0.99)	0.045
Ki67 index	1.01 (1.00-1.02)	0.028	1.03 (1.01-1.05)	0.012

^1^Cox regression model. Bold values indicate significance.

mNHERF1 membranous NHERF1, cNHERF1 cytoplasmic NHERF1, nNHERF1 nuclear NHERF1, nBRCA1 nuclear BRCA1, nPARP1 nuclear PARP1, ER estrogen receptor, PgR progesterone receptor, DFS disease free survival, OS overall survival, HR hazard ratio, CI confidence interval

No significant association was found in the multi-variate analysis for either DFS or OS using dichotomized and continuous variables of the studied proteins (Supplementary Table 3).

### Analyses in TNBC patients

In this study, 80/308 (26%) patients had TNBC. All patients were diagnosed as having IDC by pathologists. We defined TNBC when IHC for ER, PgR, and HER2/neu and FISH for HER2/neu were all negative. Moreover, 84% of patients had poorly differentiated tumors (G3), while 16% had G2 histological grade. Kaplan-Meier curves showed that patients with TNBC had a lower OS than the OS of the non TNBC patients (*p*=0.0007) (Figure 2B). Moreover, we found a lower expression of mNHERF1 (*p*=0.002) and nNHERF1 (*p*=0.006) in TNBCs, with respect to the non-TNBC tumors.

When the continuous expression data of the proteins was considered, a statistically significant association between nBRCA1 and nPARP1 expression was maintained (r_s_=0.52; *p*<0.0001), and an association between nNHERF1 and nPARP1 expression was also observed (r_s_=0.32; *p*=0.010) (Table 4). Regarding the analysed proteins, univariate analysis revealed that only the subgroup of patients with positive expression of nPARP1 and cNHERF1 had a shorter 5-year OS, 70% (95% CI:42-98) vs 92% (95% CI: 84-100) of the other patients (*p*=0.048) (Figure 2C). No statistical significance was found with multivariate analysis. The relationship between protein expression and TNBC survival was then investigated. Kaplan-Meier curves revealed that the patients with positive nuclear PARP1 expression tended toward a poorer OS than patients with negative nuclear PARP1 expression (*p*=0.072) (Figure 2D).

**Table 4:** Correlation between protein expressions in the subgroup of triple negative breast cancer (TNBC n=80)

	cNHERF1	nNHERF1	nBRCA1	nPARP1
	r_s_ (p)	r_s_ (p)	r_s_ (p)	r_s_ (p)
mNHERF1	0.07 (0.521)	0.04 (0.724)	-0.04 (0.745)	0.17 (0.180)
cNHERF1	-	0.02 (0.890)	0.02 (0.906)	0.17 (0.184)
nNHERF1	-	-	0.18 (0.160)	0.32 (0.010)
nBRCA1	-	-	-	0.52 (<0.0001)

r_s_ (p)_:_ Spearman correlation coefficient (Rho)

mNHERF1 membranous NHERF1, cNHERF1 cytoplasmic NHERF1, nNHERF1 nuclear NHERF1, nBRCA1 nuclear BRCA1, nPARP1 nuclear PARP1

## DISCUSSION

The treatment of breast cancer is based on therapeutic approaches by taking into account known biomarkers. New effective markers which help the prediction of progression risk and which target for new therapeutic treatments, would be of great benefit in breast cancer. In the present study, we examined the subcellular expression of NHERF1, BRCA1 and PARP1 proteins in invasive breast carcinomas and investigated, for the first time, the relationship among their expression and with patient outcome.

Our results show that nNHERF1 expression is associated with nBRCA1 expression. It must be noted that the majority of nBRCA1 negative tumors were simultaneously negative also for nNHERF1 expression. This is in line with our previous results, in which the unsupervised hierarchical clustering analysis showed that the low expression of nBRCA1, and low nNHERF1 expression, were associated with the group of familial patients with a more aggressive phenotype [24]. In addition, a direct correlation between cNHERF1 and nPARP1 was also demonstrated in the entire cohort of patients. It is well known that cNHERF1 expression increases gradually in breast cancer cells during carcinogenesis, and that its overexpression identifies a phenotype of breast tumors with the worst prognosis [16, 23, 39]. Moreover, the patients with high nPARP1 expression tended toward a worse DFS and had a lower median survival time [28]. These results lead us to speculate that the evaluation of cNHERF1 expression together with nPARP1 expression could be useful to stratify patients with different prognoses. However, further studies are mandatory to establish this hypothesis.

Data in the literature for ovarian cancer showed an inverse association between PARP1 and BRCA1 expression, supporting the hypothesis that loss or dysfunction of one crucial DNA repair pathway can be compensated by a “shift toward” and up-regulation of alternate DNA repair mechanisms [29, 40]. To the contrary, in the present study we found, a direct statistical correlation between nPARP1 and nBRCA1 expression, similar to that which has already been demonstrated [37, 38] and in agreement with Green et al [41]. These results suggest that the relationship between the two markers might be influenced by the location and type of tumor. Moreover, it is known that PARP1 PARylates BRCA1 and they are part of the PARP1-RAP80-BRCA1 complex, thus their interaction might be due to the activity and status of other proteins involved [42].

Regarding the clinicopathological features, in our series of breast cancers, mNHERF1 expression was positively associated with favourable clinicopathological features, such as ER-positivity, PgR-positivity and low proliferative activity. The good prognostic relevance of membranous NHERF1 is in agreement with our previous reports [43]. NHERF1 expression has been reported to be up-regulated by estrogen also in breast tumors [7, 16, 17]. In agreement with these Authors, we observed a lower expression of mNHERF1 and nNHERF1 in TNBCs, which are ER-negative tumors, with respect to the non-TNBC tumors. Moreover, during breast cancerogenesis, NHERF1 progressively becomes mostly cytoplasmic [19] and the membranous localization is lost or reduced. Consistent with Cardone *et al* [15] and Paradiso *et al* [23], here we found that cNHERF1 expression is associated with high histological grade, identifying tumors with poor prognostic features. However, we also observed an association between positive cNHERF1 expression and negative lymph node status, probably due to the presence of a higher number of patients without lymph node metastases in our series.

No statistically significant association between the nNHERF1 expression and clinicopathological variables was found, in contrast to our previous studies [23, 24] in which it was demonstrated that the loss of nNHERF1 expression in breast cancer was associated with aggressive clinical parameters and unfavourable prognosis.

In the present study, a significant statistical association between nBRCA1 and HER2/neu status was also observed. Indeed, the majority of HER2/neu positive tumors showed a negative BRCA1 expression as already demonstrated [37]. This result supports that the loss of nBRCA1 expression in breast tumors may lead to a more aggressive tumor phenotype [24]. This finding has also been confirmed by Rakha et al [44] who demonstrated that absent or reduced nBRCA1 expression was associated with other parameters of poor prognosis and with shorter DFS. As regards patient outcome, there was no association between the analyzed protein and survival status.

In comparison to our previous study [23], nNHERF1 did not confirm its role as an independent prognostic factor for survival. In fact, the patients of the previous study were enrolled into a prospective, randomized, multicenter clinical trial if they had rapidly proliferating breast cancer [45], while our cohort was heterogeneous and 48% of the patients had a low Ki67 index. Nevertheless, the univariate analysis showed that the patient subgroup with positive nNHERF1 and nPARP1 expression tended toward a shorter 5-year OS. On the basis of this result, it could be speculated that nPARP1 expression is the dominant factor influencing OS, since positive nNHERF1 expression was not previously associated with poor prognosis [23]. Further prospective investigations are required to clarify the biological role of this association and also the function of NHERF1 in the nucleus.

Triple-negative breast cancer is a specific subtype of breast cancer and it is defined clinically as lacking ER, PgR and HER2/neu. Due to the high level of heterogeneity, aggressiveness, and the lack of well-defined molecular targets, the treatment of TNBC remains a challenge [46, 47]. Also in our study, the TNBC patients had poor overall survival with respect to other BC subtypes. As demonstrated in the entire cohort, in the TNBC subgroup we also found an association between nBRCA1 and nPARP1 expression. BRCA1 is responsible for DNA repair and has been closely related to breast cancer, particularly in TNBCs [48, 49]. To date, the application of PARP inhibitors to TNBC has mainly been based on the morphologic-molecular similarities with BRCA1-mutated breast cancers and on the results from clinical studies, but not on PARP-related pathways or on the status of PARP proteins. It has been pointed out that a low or lack of expression of the target protein could misdirect the interpretation of the clinical trials with PARP inhibitors [50].

Therefore, in this context, we hypothesized that the coupled expression of nBRCA1 and nPARP1 biomarkers might be useful in selecting the optimal treatment for TNBC patients. In addition, those patients with positive nPARP1 expression had a trend towards poor OS, thus emphasizing that this biomarker could be considered of prognostic value. Moreover, in the TNBCs we found a direct correlation between nNHERF1 and nPARP1. As identification of new biomarkers for these patients is extremely important for prognosis and therapeutic purposes, we speculate that NHERF1 assessment might present a new scenario for clinical management. Although multivariate Cox regression analysis did not indicate an independent predictor of outcome, the univariate analysis showed that TNBCs with positive nPARP1 and cNHERF1 expression had a poor 5-year OS. This result highlights a group of patients with more malignant features, considering that also cNHERF1 expression was related to aggressive clinical parameters [15, 23] and the expression of these markers may have a role in the poor prognosis. This aspect could further contribute to predicting and stratifying TNBC patients and it could be important to select patients who need novel therapeutic agents. Future studies will be carried out in order to confirm this result.

In summary, NHERF1 expression could be considered a new potential biomarker in combination with PARP1 and BRCA1 expression to stratify breast cancer patients. In particular, in TNBC patients, the cytoplasmic NHERF1 associated with nuclear PARP1 expression identified a patient subgroup with a shorter survival, for whom it may be useful to identify novel target therapy strategies.

## MATERIALS AND METHODS

### Patients and clinicopathological characteristics

This study involved a retrospective, not consecutive, series of 308 patients with a diagnosis of invasive breast cancer who underwent surgery at the IRCCS Istituto Tumori “Giovanni Paolo II” of Bari between 1996 and 2012. The patients signed an informed consent form authorizing the Institute to utilize biological materials for research purpose according to ethical standards. This study was conducted in accordance with the international standards of good clinical practice.

Clinicopathological data of the patients (Supplementary Table 1), including age, histological type, tumor size, lymph node status, histological grade, ER, PgR, proliferative activity (Ki67 index) and HER2/neu status, were obtained from the Pathology Department of our Institute. ER, PgR, Ki67 and HER2/neu status were performed as previously described [23]. Tumors with ER or PgR expression were scored as positive when nuclear immunoreactivity was present in >10% of tumor cells. For the proliferative activity (Ki67 index), assessed by MIB1 nuclear staining, the cut-off value of 20% positive cells was adopted and the tumors with proliferative activity >20% were considered highly proliferating. This cut-off represents the median value of the scores relative to all breast tumor samples analysed during the last 5 years within our Institute. The HER2/neu was scored as 0, 1+, 2+ or 3+ using a monoclonal antibody (MoAb clone CB11, Novocastra Laboratories Ltd, Newcastle, UK), in accordance with the Herceptest scoring system (Food and Drug Administration accepted): 0 = no membranous immunoreactivity or <10% of cells reactive; 1+ = incomplete membranous reactivity in >10% of cells; 2+ = ≥ 10% of cells with weak to moderate complete membranous reactivity; and 3+ = strong and complete membranous reactivity in >10% of cells. Cytoplasmic immunoreactivity was ignored. Cases scoring 0 and 1+ were classified as negative. HER2/neu was considered to be positive if immunostaining was 3+ or if a 2+ result showed gene amplification by fluorescence *in situ* hybridization (FISH). In FISH analyses, each copy number of the HER2 gene and its centromere 17 (CEP17) reference was counted. The interpretation followed the criteria of the ASCO/CAP 2007 guidelines for HER2 testing in breast cancer; the cases were considered positive if the HER2/CEP17 ratio was higher than 2.2 [51].

### Clinical follow-up

Patients were routinely followed up after surgery. Follow-up was available for all patients enrolled in the present study and the median was 73 months (range 7-203 months) at the time of writing. DFS, in months, was defined as the time from diagnosis to the date of the first locoregional or distant recurrence, second invasive breast carcinoma, as well as the appearance of a second primary invasive cancer, and/or to the date of death without evidence of cancer or to the date of last visit. OS in months was defined as the time from the diagnosis to the date of death for any cause or of the last follow up. During follow up, 55 patients developed recurrence (18%) and 13 (4%) died.

### TMA construction and immunohistochemistry

NHERF1, PARP1 and BRCA1 expression patterns were examined by immunohistochemistry on TMAs containing 924 tumor tissue cores from 308 breast cancer patients. TMAs were generated using all available formalin-fixed and paraffin-embedded (FFPE) breast tumor tissue blocks. Briefly, three different regions of tumors were identified and marked on haematoxylin and eosin stained sections. Sections were matched to their corresponding paraffin blocks (donor blocks), and three tumor cores with a diameter of 1 mm were punched from these tumor regions of each donor block and precisely arrayed into a new recipient paraffin block (TMA block) using the Galileo Tissue MicroArrayer CK 4500 (Transgenomic). Each sample was arrayed in triplicate to minimize tissue loss and to overcome tumor heterogeneity. The three cores were representative of the whole tumor sample. Four μm-thick slices were cut from the TMA blocks and transferred to slides. The TMA slides were processed and stained by the indirect immunoperoxidase method using the BenchMark XT automated staining instrument (Ventana Medical Systems, Tucson, AZ,USA). All solutions were from Ventana Medical Systems unless otherwise specified. Briefly, slides underwent deparaffinization with the EZ PREP solution, followed by antigen retrieval with Cell Conditioning solution 1 (60 min, 95°C) for BRCA1 and Cell Conditioning solution 2 (36 min, 95°C) for PARP1. No antigen retrieval was executed for NHERF1. The following step was incubation with the specific primary antibody diluted in phosphate buffered saline 1X/bovine serum albumin 1% (PBS1X/BSA1%): rabbit polyclonal NHERF1 antibody (anti-EBP50; ThermoFisher Scientific, Rockford, IL, USA) at dilution 1:350 (16 min, 37°C), mouse monoclonal PARP1 antibody (F-2 clone, Santa Cruz Biotechnology Inc., Santa Cruz, CA, USA) at 1:500 dilution (16 min, 37°C), mouse monoclonal BRCA1 antibody (MS110 clone; Oncogene Research Products, Calbiochem, Merck KGaA, Darmstadt, Germany) at dilution 1:75 (32 min, 37°C). The dilution of the primary antibodies was based on preliminary dilution experiments. The UltraView Universal DAB detection kit was used to detect the protein expression. Slides were counterstained with Haematoxylin and Bluing Reagent for 8 min and 4 min, respectively. Known positive breast cancer FFPE sections were used as positive controls. For negative control, the primary antibody was omitted and replaced by PBS1X pH 7.6. Positive and negative controls were included in each staining run, as indicated in the data sheet of each antibody. The accuracy, reliability and reproducibility assessments of these antibodies (PARP1, BRCA1 and NHERF1) have been validated in studies previously published [15, 19, 23, 31, 37, 38, 52].

### NHERF1, BRCA1 and PARP1 immunohisto chemical assessment

The cores were independently evaluated for NHERF1, BRCA1 and PARP1 expression by two observers blind to patient outcome and clinicopathological data. Any discrepancies between the two observers were resolved by re-examination and consensus. Protein expression was quantified by counting the positive cells in each core on TMA at x20 magnification and expressed as a percentage of positive cells/core. Only immunostaining of invasive cancer cells within the tissue cores were considered. The mean of three readings relative to the three cores for each tumor sample was calculated and represented the protein expression of each tumor. If one core was uninformative, or either lost or contained no tumor tissue, the overall score applied was that of the remaining cores. Furthermore, the cases in which all three cores were uninformative were considered non-assessable and excluded from the analyses.

For NHERF1, the membranous, cytoplasmic and nuclear localizations were assessed in tumor cells. NHERF1 immunostaining was predominantly cytoplasmic, and in the majority of cases was positive for cNHERF1; a membranous and nuclear staining was also observed. They were scored separately and their significance was evaluated by statistical analysis. For BRCA1 and PARP1, nuclear localization was mainly observed. Cytoplasmic staining of BRCA1 and PARP1 was observed occasionally, but it was not evaluated for the purposes of the study. For mNHERF1, cNHERF1, nNHERF1 and nBRCA1 expression, the median values of tumor protein expression were considered as cut-off. As described in previous reports [23, 37], the tumors were classified as positive when the immunoreactivity of these markers was present in ≥the median value (%) or >0% if the median value was 0% (mNHERF1 >0 (median value 0), cNHERF1 ≥60.0 (median value 60.0), nNHERF1 >0 (median value 0) and nBRCA1 ≥3.0 (median value 3.0)).

nPARP1 expression was scored by the multiplicative quickscore (QS) method [37]. This system accounts for both the intensity and the extent of cell staining. The percentage of positive cells was estimated and assigned a score on a scale from 1 to 6 (1 = 1% to 4%, 2 = 5% to 19%, 3 = 20% to 39%, 4 = 40% to 59%, 5 = 60% to 79%, and 6 = 80% to 100%). The average intensity of the positive staining of cells was assigned a score from 0 to 3 (0 = no staining, 1 = weak, 2 = intermediate, and 3 = strong staining). A final QS was calculated by multiplying the percentage score by the intensity score. Based on the QS, nuclear PARP1 expression was graded as low (0-9, further referred to as negative) or high (10-18, further referred to as positive).

### Statistical analyses

In order to explore the relationship between the proteins, we performed a statistical association using both the dichotomized variables and the continuous data of protein expression. Chi-square and Fisher’s exact tests were applied to analyse the association between NHERF1, BRCA1 and PARP1 expression and clinicopathological variables (tumor size, lymph node status, histological grade, ER, PgR, Ki67 and HER2/neu status) and for statistical association between the protein expressions. Spearman’s correlation analysis was used to investigate the correlation between NHERF1, BRCA1 and PARP1 expression considered as continuous variables. The results from the immunohistochemical analyses of NHERF1, BRCA1 and PARP1 were assessed in relation to DFS and OS. Survival curves were calculated according to the Kaplan-Meier analysis and compared by the log-rank test. Univariate and multivariate Cox regression analyses were performed to estimate the hazard ratio (HR) and 95% confidence intervals (95% CIs) in order to evaluate the prognostic relevance of single protein expression (continues or dichotomized variable). All statistical differences were considered significant at a level of p<0.05. Statistical analyses were performed using SPSS software version 15 (SPSS Inc., Chicago, IL, USA).

## SUPPLEMENTARY MATERIALS TABLES







## Abbreviations

BC: breast cancer
BSA1%: bovine serum albumin 1%
CI: Confidence Intervals
cNHERF1: cytoplasmic NHERF1
DDR: DNA damage response
DFS: disease-free survival
EBP50: ezrin-radixin-moesin (ERM) binding phosphoprotein of 50 KDa
ER: estrogen receptor
FFPE: formalin-fixed and paraffin-embedded
FISH: fluorescence in situ hybridization
HER2: epidermal growth factor receptor 2
HR: Hazard ratio
IDC: invasive ductal carcinoma
IHC: immunohistochemistry
mNHERF1: membranous NHERF1
nBRCA1: nuclear BRCA1
NHERF1: Na+/H+ Exchanger Regulatory Factor 1
nNHERF1: nuclear NHERF1
nPARP1: nuclear PARP1
OS: overall survival
p: p-value
PARP1: Poly[ADP-Ribose] Polymerase 1
PBS1X: phosphate buffered saline 1X
PgR: progesterone receptor
QS: quickscore
TMA: tissue microarray
TNBC: triple negative breast cancer
