# Supplementary material for: NHERF1 together with PARP1 and BRCA1 expression as a new potential biomarker to stratify breast cancer patients
Source: Oncotarget. 2017 Jul 22;8(39):65730–42. doi: 10.18632/oncotarget.19444 (PMC5630367; doi:10.18632/oncotarget.19444)
Supplement: Supplementary file 1 [file oncotarget-08-65730-s001.pdf]

NHERF1 together with PARP1 and BRCA1 expression as a new potential biomarker to stratify breast cancer patients

SUPPLEMENTARY MATERIALS

Supplementary Table 1: Tumor clinicopathological characteristic of 308 breast cancer patients

See Supplementary File 1

Supplementary Table 2: Association between protein expression and clinicopathological characteristics

See Supplementary File 2

Supplementary Table 3: Multivariate analysis of DFS and OS (Cox regression model) in 308 patients with breast cancer

		DFS		OS	
		**HR (95% CI)**	***p-value***	**HR (95% CI)**	***p-value***
Dichotomized variables	mNHERF1	0.77 (0.30-1.97)	0.591	1.39 (0.25-7.68)	0.703
	cNHERF1	1.18 (0.57-2.46)	0.653	1.29 (0.26-6.36)	0.754
	nNHERF1	1.16 (0.42-3.22)	0.773	3.06 (0.62-15.22)	0.171
	nBRCA1	1.28 (0.61-2.68)	0.506	0.99 (0.19-5.07)	0.995
	nPARP1	0.78 (0.32-1.88)	0.586	1.71 (0.36-8.18)	0.501
Continuous data	mNHERF1	0.99 (0.96-1.02)	0.543	1.03 (0.99-1.07)	0.135
	cNHERF1	1.00 (0.99-1.02)	0.759	0.99 (0.96-1.02)	0.548
	nNHERF1	1.00 (0.96-1.04)	0.985	0.98 (0.89-1.08)	0.697
	nBRCA1	1.00 (0.98-1.03)	0.704	1.03 (0.99-1.07)	0.204
	nPARP1	0.98 (0.95-1.01)	0.255	0.99 (0.92-1.06)	0.728

mNHERF1 membranous NHERF1, cNHERF1 cytoplasmic NHERF1, nNHERF1 nuclear NHERF1, nBRCA1 nuclear BRCA1, nPARP1 nuclear PARP1, DFS disease free survival, OS overall survival, HR hazard ratio, CI confidence interval
