# Supplementary material for: NHERF1 together with PARP1 and BRCA1 expression as a new potential biomarker to stratify breast cancer patients
Source: Oncotarget. 2017 Jul 22;8(39):65730–42. doi: 10.18632/oncotarget.19444 (PMC5630367; doi:10.18632/oncotarget.19444)
Supplement: Supplementary file 2 [file oncotarget-08-65730-s002.doc]

Supplementary Table 1: Tumor clinicopathological characteristic of 308 breast cancer patients
Characteristics	n	(%)	
			
Patients Age: median value 51 (range 24-80)			
≤ 51 years	158	(51.3)	
> 51 years	150	(48.7)	
			
Histological type			
IDC	268	(87.0)	
ILC	23	(7.5)	
Other	17	(5.5)	
			
Histological grade			
G1	22	(7.2)	
G2	144	(47.4)	
G3	138	(45.4)	
Unknown 	4		
			
Tumor size (cm)			
≤2 cm	156	(53.8)	
>2 cm	134	(46.2)	
Unknown	18		
			
Lymph node status			
Negative	174	(57.2)	
Positive	130	(42.8)	
Unknown 	4		
			
Receptor status			
ER-negative (≤10%)	110	(35.8)	
ER-positive (>10%)	197	(64.2)	
Unknown 	1		
			
PgR-negative (≤10%)	147	(47.9)	
PgR-positive (>10%)	160	(52.1)	
Unknown 	1		
			
Ki67 index			
Negative (≤20%)	147	(48.0)	
Positive (>20%)	159	(52.0)	
Unknown	2		
			
HER2/neu 			
Negative (0,1+) 	241	(81.7)	
Positive  (3+)	54	(18.3)	
Unknown	13		
			
Treatment			
CT	98	(32.7)	
HT	83	(27.7)	
CT+HT	119	(39.6)	
Unknown 	8		
			
TNBC 	80/308	(26.0)	
			

        IDC invasive ductal carcinoma, ILC invasive lobular carcinoma, ER estrogen receptor, PgR progesterone receptor TNBC, triple negative breast cancer
