# Supplementary material for: NHERF1 together with PARP1 and BRCA1 expression as a new potential biomarker to stratify breast cancer patients
Source: Oncotarget. 2017 Jul 22;8(39):65730–42. doi: 10.18632/oncotarget.19444 (PMC5630367; doi:10.18632/oncotarget.19444)
Supplement: Supplementary file 3 [file oncotarget-08-65730-s003.docx]

**Supplementary Table 2: Association between protein expression and clinicopathological characteristics**

|  |  | *mNHERF1* | | | |  |  | *cNHERF1* | | | |  |  | *nNHERF1* | | | |  |  | *nBRCA1* | | | |  |  | *nPARP1* | | | |  |
| --- | --- | --- | --- | --- | --- | --- | --- | --- | --- | --- | --- | --- | --- | --- | --- | --- | --- | --- | --- | --- | --- | --- | --- | --- | --- | --- | --- | --- | --- | --- |
|  |  | Negative | | Positive | |  |  | Negative | | Positive | |  |  | Negative | | Positive | |  |  | Negative | | Positive | |  |  | Negative | | Positive | |  |
| Characteristics |  | n | (%) | n | (%) | *p-value* |  | n | (%) | n | (%) | *p-value* |  | n | (%) | n | (%) | *p-value* |  | n | (%) | n | (%) | *p-value* |  | n | (%) | n | (%) | *p-value* |
|  |  |  |  |  |  |  |  |  |  |  |  |  |  |  |  |  |  |  |  |  |  |  |  |  |  |  |  |  |  |  |
| *Patient age* |  |  |  |  |  |  |  |  |  |  |  |  |  |  |  |  |  |  |  |  |  |  |  |  |  |  |  |  |  |  |
| ≤ 51 years |  | 111 | (52.1) | 31 | (45.6) | *NS* |  | 76 | (61.8) | 66 | (41.8) | *0.0009* |  | 123 | (52.3) | 19 | (41.3) | *NS* |  | 61 | (48.8) | 69 | (52.7) | *NS* |  | 94 | (50.3) | 37 | (48.7) | *NS* |
| > 51 years |  | 102 | (47.9) | 37 | (54.4) |  |  | 47 | (38.2) | 92 | (58.2) |  |  | 112 | (47.7) | 27 | (58.7) |  |  | 64 | (51.2) | 62 | (47.3) |  |  | 93 | (49.7) | 39 | (51.3) |  |
|  |  |  |  |  |  |  |  |  |  |  |  |  |  |  |  |  |  |  |  |  |  |  |  |  |  |  |  |  |  |  |
| *Histological type* |  |  |  |  |  |  |  |  |  |  |  |  |  |  |  |  |  |  |  |  |  |  |  |  |  |  |  |  |  |  |
| IDC |  | 189 | (88.7) | 58 | (85.3 | *NS* |  | 110 | (89.4) | 137 | (86.7) | *NS* |  | 206 | (87.7) | 41 | (89.1) | *NS* |  | 113 | (90.4) | 112 | (85.5) | *NS* |  | 173 | (92.6) | 59 | (77.6) | *0.0060* |
| ILC |  | 14 | (6.6) | 4 | (5.9) |  |  | 5 | (4.1) | 13 | (8.2) |  |  | 18 | (7.6) | 0 | (0.0) |  |  | 6 | (4.8) | 12 | (9.2) |  |  | 7 | (3.7) | 12 | (15.8) |  |
| Other |  | 10 | (4.7) | 6 | (8.8) |  |  | 8 | (6.5) | 8 | (5.1) |  |  | 11 | (4.7) | 5 | (10.9) |  |  | 6 | (4.8) | 7 | (5.3) |  |  | 7 | (3.7) | 5 | (6.6) |  |
|  |  |  |  |  |  |  |  |  |  |  |  |  |  |  |  |  |  |  |  |  |  |  |  |  |  |  |  |  |  |  |
| *Tumor size (cm)* |  |  |  |  |  |  |  |  |  |  |  |  |  |  |  |  |  |  |  |  |  |  |  |  |  |  |  |  |  |  |
| ≤2 cm |  | 97 | (49.0) | 39 | (60.0) | *NS* |  | 62 | (53.2) | 74 | (50.3) | *NS* |  | 111 | (50.9) | 25 | (55.6) | *NS* |  | 58 | (47.9) | 66 | (55.5) | *NS* |  | 95 | (52.8) | 34 | (50.7) | *NS* |
| >2 cm |  | 101 | (51.0) | 26 | (40.0) |  |  | 54 | (46.5) | 73 | (49.7) |  |  | 107 | (49.1) | 20 | (44.4) |  |  | 63 | (52.1) | 53 | (44.5) |  |  | 85 | (47.2) | 33 | (49.3) |  |
|  |  |  |  |  |  |  |  |  |  |  |  |  |  |  |  |  |  |  |  |  |  |  |  |  |  |  |  |  |  |  |
| *Lymph node status* |  |  |  |  |  |  |  |  |  |  |  |  |  |  |  |  |  |  |  |  |  |  |  |  |  |  |  |  |  |  |
| Negative |  | 112 | (53.6) | 44 | (64.7) | *NS* |  | 60 | (49.6) | 96 | (61.5) | *0.0470* |  | 130 | (56.0) | 26 | (57.8) | *NS* |  | 73 | (58.9) | 66 | (51.2) | *NS* |  | 104 | (55.9) | 41 | (55.4) | *NS* |
| Positive |  | 97 | (46.4) | 24 | (35.3) |  |  | 61 | (50.4) | 60 | (38.5) |  |  | 102 | (44.0) | 19 | (42.2) |  |  | 51 | (41.1) | 63 | (48.8) |  |  | 82 | (44.1) | 33 | (44.6) |  |
|  |  |  |  |  |  |  |  |  |  |  |  |  |  |  |  |  |  |  |  |  |  |  |  |  |  |  |  |  |  |  |
| *Histological grade* |  |  |  |  |  |  |  |  |  |  |  |  |  |  |  |  |  |  |  |  |  |  |  |  |  |  |  |  |  |  |
| G1 |  | 13 | (6.2) | 6 | (9.0) | *NS* |  | 13 | (10.6) | 6 | (3.9) | *0.0460* |  | 16 | (6.9) | 3 | (6.5) | *NS* |  | 9 | (7.3) | 9 | (7.0) | *NS* |  | 14 | (7.5) | 2 | (2.7) | *NS* |
| G2 |  | 92 | (43.8) | 34 | (50.7) |  |  | 57 | (46.3) | 69 | (44.8) |  |  | 100 | (43.3) | 26 | (56.5) |  |  | 52 | (41.9) | 64 | (50.0) |  |  | 87 | (46.8) | 36 | (49.3) |  |
| G3 |  | 105 | (50.0) | 27 | (40.3) |  |  | 53 | (43.1) | 79 | (51.3) |  |  | 115 | (49.8) | 17 | (37.0) |  |  | 63 | (50.8) | 55 | (43.0) |  |  | 85 | (45.7) | 35 | (48.0) |  |
|  |  |  |  |  |  |  |  |  |  |  |  |  |  |  |  |  |  |  |  |  |  |  |  |  |  |  |  |  |  |  |
| *Receptor status* |  |  |  |  |  |  |  |  |  |  |  |  |  |  |  |  |  |  |  |  |  |  |  |  |  |  |  |  |  |  |
| ER-negative (≤ 10%) |  | 93 | (43.9) | 12 | (17.6) | *0.0001* |  | 47 | (38.2) | 58 | (36.9) | *NS* |  | 84 | (35.9) | 21 | (45.6) | *NS* |  | 39 | (31.2) | 48 | (36.9) | *NS* |  | 64 | (34.2) | 26 | (34.7) | *NS* |
| ER-positive (>10%) |  | 119 | (56.1) | 56 | (82.4) |  |  | 76 | (61.8) | 99 | (63.1) |  |  | 150 | (64.1) | 25 | (54.4) |  |  | 86 | (68.8) | 82 | (63.1) |  |  | 123 | (65.8) | 49 | (65.3) |  |
|  |  |  |  |  |  |  |  |  |  |  |  |  |  |  |  |  |  |  |  |  |  |  |  |  |  |  |  |  |  |  |
| PgR-negative (≤ 10%) |  | 117 | (55.2) | 24 | (35.3) | *0.0040* |  | 57 | (46.3) | 84 | (53.5) | *NS* |  | 116 | (49.6) | 25 | (54.4) | *NS* |  | 58 | (46.4) | 63 | (48.5) | *NS* |  | 89 | (47.6) | 37 | (49.3) | *NS* |
| PgR-positive (>10%) |  | 95 | (44.8) | 44 | (64.7) |  |  | 66 | (53.7) | 73 | (46.5) |  |  | 118 | (50.4) | 21 | (45.6) |  |  | 67 | (53.6) | 67 | (51.5) |  |  | 98 | (52.4) | 38 | (50.7) |  |
|  |  |  |  |  |  |  |  |  |  |  |  |  |  |  |  |  |  |  |  |  |  |  |  |  |  |  |  |  |  |  |
| *Ki67 index* |  |  |  |  |  |  |  |  |  |  |  |  |  |  |  |  |  |  |  |  |  |  |  |  |  |  |  |  |  |  |
| Negative (≤ 20%) |  | 87 | (41.2) | 38 | (55.9) | *0.0350* |  | 53 | (43.4) | 72 | (45.9) | *NS* |  | 105 | (45.1) | 20 | (43.5) | *NS* |  | 55 | (44.0) | 62 | (47.7) | *NS* |  | 85 | (45.5) | 36 | (48.0) | *NS* |
| Positive (> 20%) |  | 124 | (58.8) | 30 | (44.1) |  |  | 69 | (56.6) | 85 | (54.1) |  |  | 128 | (54.9) | 26 | (56.5) |  |  | 70 | (56.0) | 68 | (52.3) |  |  | 102 | (54.5) | 39 | (52.0) |  |
|  |  |  |  |  |  |  |  |  |  |  |  |  |  |  |  |  |  |  |  |  |  |  |  |  |  |  |  |  |  |  |
| *HER2/neu* |  |  |  |  |  |  |  |  |  |  |  |  |  |  |  |  |  |  |  |  |  |  |  |  |  |  |  |  |  |  |
| Negative (0, 1+) |  | 162 | (78.3) | 58 | (89.2) | *0.0500* |  | 96 | (81.4) | 124 | (80.5) | *NS* |  | 183 | (79.9) | 37 | (86.1) | *NS* |  | 90 | (73.8) | 105 | (84.7) | *0.0350* |  | 143 | (79.4) | 58 | (80.6) | *NS* |
| Positive (3+) |  | 45 | (21.7) | 7 | (10.8) |  |  | 22 | (18.6) | 30 | (19.5) |  |  | 46 | (20.1) | 6 | (13.9) |  |  | 32 | (26.2) | 19 | (15.3) |  |  | 37 | (20.6) | 14 | (19.4) |  |
|  |  |  |  |  |  |  |  |  |  |  |  |  |  |  |  |  |  |  |  |  |  |  |  |  |  |  |  |  |  |  |

*p-value* by Chi-square or Fisher Test. Bold values indicate significance

NS not significant, IDC invasive ductal carcinoma, ILC invasive lobular carcinoma, mNHERF1 membranous NHERF1, cNHERF1 cytoplasmic NHERF1, nNHERF1 nuclear NHERF1, nBRCA1 nuclear BRCA1, nPARP1 nuclear PARP1, ER estrogen receptor, PgR progesterone receptor
